# The Detection of the Methylated Wif-1 Gene Is More Accurate than a Fecal Occult Blood Test for Colorectal Cancer Screening

**DOI:** 10.1371/journal.pone.0099233

**Published:** 2014-07-15

**Authors:** Aurelien Amiot, Hicham Mansour, Isabelle Baumgaertner, Jean-Charles Delchier, Christophe Tournigand, Jean-Pierre Furet, Jean-Pierre Carrau, Florence Canoui-Poitrine, Iradj Sobhani, for the CRC group of Val De Marne

**Affiliations:** 1 Department of Gastroenterology, APHP, Henri-Mondor hospital, Créteil, France; 2 University of Paris Est Creteil, Créteil, France; 3 Laboratoire d'Investigation Clinique EA 4393 EC2M3, Créteil, France; 4 Bioscience Core Laboratories, King Abdullah University of Science and Technology, Thuwal, Saudi Arabia; 5 Department of Oncology, APHP, Henri-Mondor Hospital, Créteil, France; 6 Commensals and Probiotics-Host Interactions Laboratory, INRA, MICALIS Institute, Jouy en Josas, France; 7 Laboratoire d'analyse, CPAM, Paris, France; 8 Department of Public Health, APHP, Henri-Mondor Hospital, Créteil, France; The Chinese University of Hong Kong, Hong Kong

## Abstract

**Background:**

The clinical benefit of guaiac fecal occult blood tests (FOBT) is now well established for colorectal cancer screening. Growing evidence has demonstrated that epigenetic modifications and fecal microbiota changes, also known as dysbiosis, are associated with CRC pathogenesis and might be used as surrogate markers of CRC.

**Patients and Methods:**

We performed a cross-sectional study that included all consecutive subjects that were referred (from 2003 to 2007) for screening colonoscopies. Prior to colonoscopy, effluents (fresh stools, sera-S and urine-U) were harvested and FOBTs performed. Methylation levels were measured in stools, S and U for 3 genes (Wif1, ALX-4, and Vimentin) selected from a panel of 63 genes; Kras mutations and seven dominant and subdominant bacterial populations in stools were quantified. Calibration was assessed with the Hosmer-Lemeshow chi-square, and discrimination was determined by calculating the C-statistic (Area Under Curve) and Net Reclassification Improvement index.

**Results:**

There were 247 individuals (mean age 60.8±12.4 years, 52% of males) in the study group, and 90 (36%) of these individuals were patients with advanced polyps or invasive adenocarcinomas. A multivariate model adjusted for age and FOBT led to a C-statistic of 0.83 [0.77–0.88]. After supplementary sequential (one-by-one) adjustment, Wif-1 methylation (S or U) and fecal microbiota dysbiosis led to increases of the C-statistic to 0.90 [0.84–0.94] (p = 0.02) and 0.81 [0.74–0.86] (p = 0.49), respectively. When adjusted jointly for FOBT and Wif-1 methylation or fecal microbiota dysbiosis, the increase of the C-statistic was even more significant (0.91 and 0.85, p<0.001 and p = 0.10, respectively).

**Conclusion:**

The detection of methylated Wif-1 in either S or U has a higher performance accuracy compared to guaiac FOBT for advanced colorectal neoplasia screening. Conversely, fecal microbiota dysbiosis detection was not more accurate. Blood and urine testing could be used in those individuals reluctant to undergo stool testing.

## Introduction

Colorectal cancer (CRC) is a significant cause of morbidity and mortality in developed countries. The incidence of CRC has grown by 3 to 4% per year since the 1970s [1], [2]. In most cases, the natural history of CRC includes a progression from polyps to advanced CRC that allows for early detection in asymptomatic patients. The screening colonoscopy, the “gold-standard method”, flexible sigmoidoscopy, and computed tomography colonography have been shown to be effective for CRC screening by demonstrating a reduction in CRC incidence and mortality [3], [4], [5], [6]. However in most countries, none of those methods are currently recommended for CRC mass screening due to the inherent limitations, which include bowel preparation, high cost, low adherence rates, and occasional adverse events. In recent decades, attempts have been made to set up minimally invasive tests for CRC screening. The guaiac fecal occult blood test (FOBT) has been shown to reduce CRC-related mortality in three randomized controlled trials and one population-based study [7], [8], [9], [10]. However, FOBT has a low sensitivity and a very limited impact on the adenoma-carcinoma transition, most likely because early precancerous lesions only produce low concentrations of blood in stools [11], [12]. The quantitative immunochemical FOBT has recently demonstrated a higher sensitivity in the detection of advanced neoplasias [13]. However, there is still a need for a more accurate test to improve screening performance.

In the majority of cases, inherited genetic factors make a minor contribution to the susceptibility to develop CRC, while the major contributors are environmental factors [14]. Indeed, previous studies have demonstrated that CRC development was associated with aging but also with modern western lifestyles and dietary components [1], [15].

The aberrant methylation of CpG-rich sequences (CpG islands) is an epigenetic change that induces the transcriptional silencing of tumor suppressor genes [16]. These gene alterations can be induced by chemical and/or environmental factors [1]. Hypermethylation of CpG islands in tumor suppressor genes has been reported in the neoplastic tissue of CRC patients, as well as in premalignant lesions [17]. It is known that aberrant methylation exists in circulating DNA, such as that found in stool, serum, urine, and other body fluids, and could be used as a biomarker for cancer screening [18], [19].

More recently, microbes colonizing the GI tract, which are major actors in biological environments, have been suspected of playing roles in the occurrence of CRC [20]. Indeed, changes in the luminal or adherent intestinal microbiota, so-called dysbiosis, have been shown in CRC patients [21], [22]. In animal models, the contribution of bacteria to the etiology of CRC has been studied, and it has been suggested that they might act through inflammatory pathways, although a direct genotoxic effect of bacteria could also be suspected [23]. Recently, we demonstrated that stool transplantation from human patients with CRC to germ-free mice could induce increases intestinal precancerous events through the host gene alteration, cell proliferation and metabolism in the mucosa [24]. Therefore, changes in the fecal microbiota could be used as biomarkers for CRC screening.

In the present study, we investigate the performance accuracy of new screening tests for CRC targeting the methylation levels of a panel of 63 genes in stools, blood, and urine, and the composition of fecal microbiota was assessed by qPCR. The latter tests were compared to guaiac FOBT in a hospital-based population of subjects belonging to groups at average or high risk for CRC.

## Patients and Methods

### Study population

From 2003 to 2007, 247 consecutive out-patients (mean age 60.8±12.4, 129 males) with an average or high risk of CRC (history of cancer in relatives, personal past history of polyps, any abdominal or intestinal related symptoms, or anemia that required colonoscopy), were included in a sample bank collection study. To be eligible for inclusion, the patients had to have no previous history of the following: colorectal surgery, diseases such as inflammatory or infectious injuries of the intestine, or a need for an emergency colonoscopy. The study took place in two departments (Gastroenterology and Oncology) of a teaching hospital in the Paris area of France.

Two weeks prior to the colonoscopy and after giving written informed consent, patients were included in the study and were asked to give fresh stool, urine and blood samples within 2 weeks but up to three days prior to the colonoscopy. In all cases, stool samples were collected prior to bowel cleansing for colonoscopy. Any particular diets (diabetics, vegetarians) and medications (anti-diabetic drugs, hypocholesterolemics, and laxatives) during this period were recorded. The study was approved by the ethical committee of the Val de Marne Paris-EST area that authorized the enrollment of patients in all associated centers (number 2004-4 CCPPRB). All patients received information about the study, its aims, and samples they would give. All information was given by a typed letter written in French, and formal written consent was obtained on a triplicate copy form; one copy of the form was retained by the patient, we retained one copy at the department of clinical research (CIC) and the last copy was retained by the promoter (National institute of scientific research in medicine-INSERM). Thus, a formal consent was available for each patient. Detailed interviews for medical history and physical examinations were performed.

### Guaiac fecal occult blood test

The guaiac Hemoccult test was performed (performed in a certificated laboratory by French authorities in Paris) on all subjects agreeing to join the study. For the FOBT, 1 sample per stool from 3 consecutive bowel movements was required. In accordance with the ongoing program, a specific diet (meatless for 3 days) was recommended. Subjects collected the 3 samples themselves at home (using the kit card for Hemoccult), recorded the date of each bowel movement involved, and sent the tests by mail. The Hemoccult reading was performed blindly by certified technicians without rehydration, according to the protocols set by the national organized screening program. Screening was considered positive if the Hemoccult test was positive (at least 1 of the 6 slots was positive).

### Screening colonoscopy

All individuals underwent a colonoscopy examination under anesthesia. Right and left colon sites, as well as the rectum, were fully examined in all patients. The colonoscopy results were recorded following the usual process for an organized screening program in France. Polyps were completely removed during endoscopy examination using mucosectomy, if required. The colonoscopy was considered to be the gold standard method. The colonoscopy findings were classified according to the most advanced pathologic lesion found. Advanced neoplasms included advanced adenomas (adenomas measuring >10 mm or adenomas with high-grade dysplasia or in situ carcinoma) and invasive CRC (invasion of malignant cells beyond the muscularis mucosae). Hyperplasic polyps were not included as neoplasms.

### Quantitation of methylation by quantitative PCR

Total DNA was isolated from urine, serum and stool samples using the Qiamp DNA Mini kit (Qiagen) and then converted with sodium bisulfite and amplified using locus-specific PCR primers flanking a MGB probe. The methylation level for each patient was determined as described by Eads et al [25]. The PCR conditions were 94°C for 10 minutes for enzyme activation, 45 cycles of 94°C for 30 seconds and 60°C for 60 seconds for annealing and elongation PCR. We confirmed methylation results by direct bisulfite genomic sequencing in 5% of affected patients investigated for their tissues to exclude a technical artifact. A panel of 63 genes (data not shown) extracted from the literature was first evaluated, and then 3 genes of interest (Wif-1, ALX-4 and Vimentin) were selected based on their discriminative values that were determined by comparisons between tumor and normal homologous intestinal tissues [26]. The efficiency of the primers of the target genes (Wif1, ALX4 and Vimentin) and the housekeeping gene (Albumin BSP) for the input of modified DNA was evaluated using serial dilutions of methylated modified DNA controls as shown in **Figure S1**). After a feasibility set of experiments performed on 48 patients' stool, urine and serum samples, we decided to measure only Wif-1 methylation levels in the validation set according to the higher sensitivity and specificity rates of the Wif-1 gene compared to ALX-4 and Vimentin. The primer and probe sequences for methylation of target genes are listed in **Table S1**.

### Bacterial analysis of fecal samples using quantitative PCR

Whole fresh stools were collected in sterile boxes, and, within 4 hours, 10 g were frozen at −80°C for analysis. Bacterial DNA was extracted from aliquots of feces, and after the final precipitation, DNA was resuspended in 150 µL of TE buffer and stored at −80°C for further analysis, as previously described [26]. We used a real-time qPCR technique to investigate the difference in bacterial densities within the microbiota between normal and advanced neoplasia and cancer patients' stools. The primers and probes used in this study have been described elsewhere [26] and are presented in **Table S2**. Real-time qPCR was performed using an ABI 7000 Sequence Detection System with software version 1.2.3 (Applied-Biosystems, Foster City, Ca, USA), and total numbers of bacteria were inferred from the averaged standard curves and expressed as log10 values, as previously described [26]. qPCR values were obtained per patient and for each gut microbiota component. To compensate for the fact that the fecal samples might contain more or less water, the data for each fecal sample were normalized as previously described [26]. The level found for each particular dominant and sub-dominant bacterial population was subtracted from the all-bacteria content, and the results are expressed as the log of the number of bacteria per gram of stool. These assays were used to compare the composition of the intestinal microbiota of all subjects, and the results are expressed as means ± SD.

### KRAS mutation analysis

DNA was amplified on duplicates by PCR with an allele-specific primer set covering exons 12 and 13 of the Kras gene [27]. The PCR conditions were 94°C for 10 minutes, 45 cycles of 94°C for 30 seconds and 60°C for 60 seconds for annealing and elongation PCR. Two sets of PCR primers and probes were used: one set was specific for wild allele DNA and the second set represented the mutation allele. The primer and probe sequences are listed in **Table S3**.

### Statistical analysis

Each patient's characteristics are described as a number (%) for qualitative data and as a median (interquartile range, IQR) or mean (±1 standard deviation, SD) for quantitative data according to their distribution. We compared the baseline characteristics of the patients of both groups (advanced neoplasia and control groups) using the Pearson chi-square test or Fisher's exact test for qualitative variables and the Wilcoxon non parametric or Kruskal- Wallis tests for quantitative variables. Spearman's two-tailed test was used to assess correlations.

The odds ratios (OR) and 95% confidence intervals (CIs) of each parameter were estimated using separate logistic regression analyses. The fecal microbiota dysbiosis results were given for 1 SD of the log-transformed levels calculated for each control group. The analysis was systematically age-adjusted to estimate independently the performance accuracy of each screening tests. Age was handled in continuous after verifying normality assumption. Calibration of each model was estimated by the Hosmer-Lemeshow statistic in which a *p* value greater than 0.20 indicates adequate fit. The discrimination of our model was assessed by the C-statistic (Area Under ROC Curve), which was calculated from the multivariate logistic regression model. The C-statistics of the models with and without advanced neoplasia were compared using a nonparametric test developed by Delong et al and appropriate for non-independent data [28]. A resampling bootstrap analysis with 1000 replications of baseline dataset was performed to estimate 95% CIs of C-statitistics of the multivariate models. The discrimination was also quantified by calculating the Net Reclassification Improvement index (NRI) [29].

All comparisons were 2-sided and a *p* value of less than 0.05 indicated a statistically significant difference. Analyses were performed using STATA software version 12.0 (Stata- Corp, College Station, TX, USA).

## Results

### Study population

Two hundred forty-seven patients were included in the study. The characteristics of the study population are listed in **Table 1**. All individuals underwent a colonoscopy with full examination of the right and left colon and the rectum. The patients were then classified as follows: normal individuals who presented with either normal colonoscopy (n = 123) or small adenomas less than 1 cm in diameter (n = 34) were herein considered to be controls and patients with neoplasia who presented with CRC (n = 66) or large (>1 cm in diameter) adenomas (n = 24) were herein considered to be cases. The average case was more than 10 years older than the average control patient. The control patients less frequently reported a previous personal history of polyps and colorectal cancer in their family compared to the cases. The two groups were equally balanced for BMI, comorbidities, medicine uptake, and food regimen (Table 1).

**Table 1:** Characteristics of the individuals included in the current study (n = 247) according to the colonoscopy and pathology results.

	Control subjects (Normal colonoscopy and small adenoma)	Cases (advanced neoplasia*)	*P†*
n	157	90	
Age, years, mean ± SD	56.6±11.3	68.3±10.5	<0.001
Male gender, n (%)	76 (48%)	53 (59%)	0.10
BMI, kg/m^2^, mean ± SD	24.9±7.1	25.8±9.5	0.39
Past history			
Polyps	40 (25%)	13 (14%)	0.05
Colorectal cancer	5 (3%)	6 (7%)	0.20
Familial history			
Polyps in First degree relatives	26 (17%)	11 (12%)	0.38
CRC inn First degree relatives	91 (58%)	30 (35%)	<0.001
Comorbidity			
Diabetes, n (%)	17 (11%)	16 (18%)	0.12
Hypercholesterolemia, n (%)	42 (27%)	30 (33%)	0.26
Any longterm treatment, n (%)	127 (80%)	73 (81%)	0.90
Particular nutriment‡			
Diabetes regimen, n (%)	13 (8%)	14 (16%)	0.08
Any others, n (%)	26 (17%)	21 (24%)	0.16
Reason for colonoscopy			
Screening	54 (34%)	17 (19%)	0.001
Symptoms	69 (44%)	62 (69%)	
History of polyps	34 (22%)	11 (12%)	

*Advanced neoplasia: advanced neoplasia included advanced adenomas (adenomas measuring >10 mm or adenomas with high-grade dysplasia, or in situ carcinoma) and invasive CRC (invasion of malignant cells beyond the muscularis mucosae); † Pearson chisquare or Fisher tests or Student Test or Wilcoxon tests as appropriate; ‡ includes those individuals who are under any particular regimen (diabetes, vegetarian, hyper proteic, hyper vitaminic etc…); ^⊥^No antibiotics;

### Analysis of Guaiac-based Fecal Occult Blood Test Hemoccult

The Hemoccult test was not interpretable in 2 cases. Overall, the FOBT was positive in 46 patients of whom 36 (78%) had advanced neoplasia, including 33 (72%) CRCs. Among the 199 patients with negative FOBTs, 146 (73%) patients had normal colonoscopies or small adenomas whereas 53 (27%) had advanced neoplasia, including 33 (17%) with CRC. Thus, the sensitivity and specificity of the FOBT for advanced neoplasms were 40.4% and 93.6%, respectively.

### Methylated Wif-1, ALX-4, and Vimentin in serum, urine, and stool

**Figure 1:**
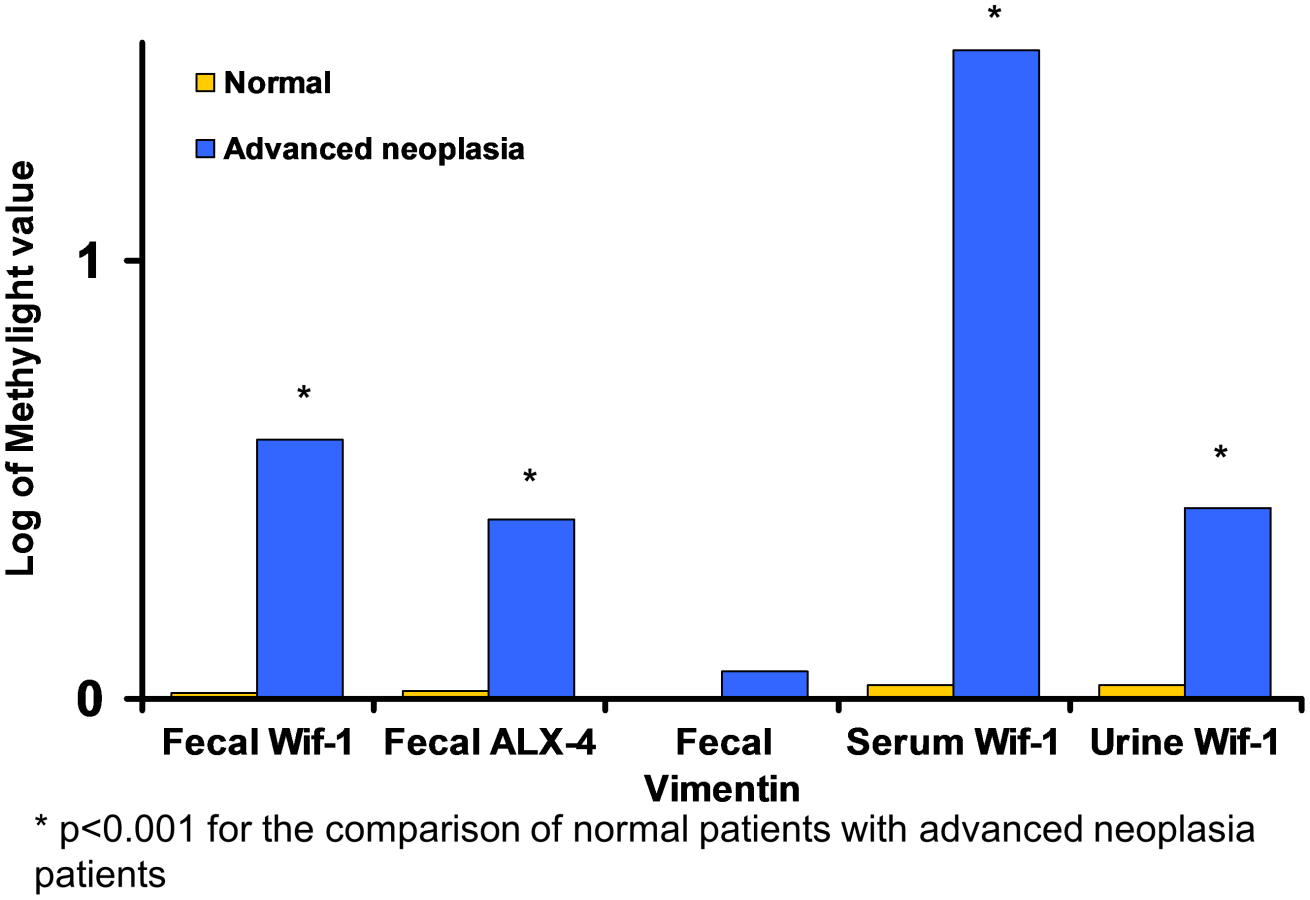
Methylation quantification according to the value detected by MethyLight assay using methylated gene-targeted primers (Wif-1, ALX-4, and Vimentin) and various effluents: stool (A), urine (B), and serum (C).

The methylation levels of the Wif-1, ALX-4 and Vimentin genes were first assessed in fecal samples. In the overall population, hypermethylation of Wif-1, ALX-4 and Vimentin genes was observed in 7.3%, 4.5% and 0.8%, respectively (Table 2). For each gene (Wif-1, ALX-4 and Vimentin), aberrant methylation was significantly more frequent in case patients compared to control patients (19.3% *vs.* 0.6%, 10.6% *vs.* 1.3% and 32.6% *vs*. 0.0%, respectively) (Table 2). For each gene, the specificity was above 98% but with a low sensitivity below 25%. For the Wif-1 and ALX-4 genes, but not the Vimentin gene, the methylation level was significantly more elevated in case patients compared to control individuals (Figure 1).

**Table 2:** Methylation levels of WIf-1, ALX-4, and Vimentin in healthy and advanced colorectal neoplasia patients.

	Overall population	Control subjects (Normal colonoscopy and small adenoma)	Cases (advanced neoplasia*)	*P†*	*Sensitivity*	*Specificity*
N	247	157	90			
Stool samples						
Wif-1	18 (7.3%)	1 (0.6%)	17 (19.3%)	<0.001	19%	99%
ALX-4	11 (4.5%)	2 (1.3%)	9 (10.6%)	0.001	11%	99%
Vimentin	2 (0.8%)	0 (0%)	29 (32.6%)	0.05	33%	100%
Serum samples						
Wif-1	31 (12.6%)	2 (1.3%)	29 (32.6%)	<0.001	33%	99%
ALX-4	11/62 (18%)	0/14 (0%)	11/48 (23%)	0.06	23%	100%
Vimentin	2/62 (3%)	0/14 (0%)	2/48 (4%)	0.99	4%	100%
Urine samples						
Wif-1	26 (10.5%)	2 (1.3%)	24 (26.7%)	<0.001	27%	99%
ALX-4	7/62 (11%)	0/14 (0%)	7/48 (15%)	0.33	15%	100%
Vimentin	4/62 (7%)	0/14 (0%)	4/48 (8%)	0.57	8%	100%
Serum or urine samples						
Wif-1	47% (19%)	4 (2.5%)	43 (47.8%)	<0.001	48%	99%

n: represents the numbers of aptients; *Advanced neoplasia: advanced neoplasia included advanced adenomas (adenomas measuring >10 mm or adenomas with high-grade dysplasia, or in situ carcinoma) and invasive CRC (invasion of malignant cells beyond the muscularis mucosae); *†*Pearson chisquare or Fisher tests as appropriate.

The methylation levels of Wif-1, ALX-4, and Vimentin were then assessed in urine and serum samples. Wif-1 appeared to be the most sensitive marker of advanced neoplasia in either urine or serum samples (52% and 29%) compared to ALX-4 and Vimentin (23% and 15% vs. 4% and 8%, respectively). Specificity was higher than 93% in all cases. The combination of urine and serum WIf-1 methylation level led to a higher sensitivity rate of 60.4% [30].

The methylation level of Wif-1 was then assessed in either urine or serum samples for the entire validation cohort, which included 247 patients. Hypermethylation of Wif-1 was observed in the urine and serum samples in 26.7% and 32.6% of the cases and in 1.3% and 1.3% of the control individuals, respectively (p<0.001 and p<0.001). The combination of serum and urine raised the neoplasia detection rate to 47.8% in cases, compared to 2.5% in controls (Table 2). The methylation level was significantly more elevated in cases compared to controls in either serum or urine samples (Figure 1). No difference was found for different stages of colorectal cancer according to the American Joint Committee on Cancer classification.

### Fecal microbiota dysbiosis in advanced adenomas (>10 mm) and colorectal cancer

The fecal microbiota composition was available for all of the subjects and is presented in Table 3. In the case patients, fecal microbiota dysbiosis was characterized by a higher density of species belonging to the *Bacteroides/Prevotella* group vs. controls. Considering all individuals together, the *Bacteroides/Prevotella* group was not linked to age (r = 0.09; p = 0.62) or BMI (r = 0.20 and p = 0.24).

**Table 3:** Composition of microbiota for dominant and sub dominant bacteria groups in healthy and advanced colorectal neoplasia patients.

	Control subjects (Normal colonoscopy and small adenoma)	Case (advanced neoplasia*)	*P†*
N	157	90	
All bacteria^⊥^	11.90±0.40	11.80±0.49	0.09
*Escherichia coli* species^‡^	−3.84±1.35	−3.57±1.27	0.14
*Bifidobacterium* genus^‡^	−2.09±1.21	−1.96±1.09	0.43
*Lactobacillus/leuconostoc/Pediococcus* ^‡^	−2.35±1.00	−2.26±0.78	0.46
*Blautia coccoides* group^‡^	−1.27±0.48	−1.25±0.43	0.80
*Clostridium leptum* group^‡^	−0.01±0.02	0.01±0.02	0.06
*Faecalibacterium prauznitzii* species^‡^	−1.03±0.87	−0.94±0.97	0.468
*Bacteroides/Prevotella* group^‡^	−1.40±0.79	−1.09±0.64	0.003

N: represents the numbers of patients; *Advanced neoplasia: advanced neoplasia included advanced adenomas (adenomas measuring >10 mm or adenomas with high-grade dysplasia, or in situ carcinoma) and invasive CRC (invasion of malignant cells beyond the muscularis mucosae); *†*

Comparison of continuous variables was performed using Student T-test or Wilcoxontest whenever appropriate; ^⊥^All-bacteria results obtained by qPCR were expressed as mean of the log10 value ± SD, ^‡^Results were expressed as mean of normalized values ± SD, calculated as the log number of targeted bacteria minus the log number of all-bacteria. *Faecalibacterium prausnitzii* is the major component of *Clostridium leptum* group.

### Kras mutation analysis in stool samples

Kras mutations (exon 12 and 13) in stool samples were found in 16 (18%) patients with advanced neoplasia compared to 16 (10%) individuals in the control group. The sensitivity and specificity were 17.8% and 89.8% for b Kras mutations, respectively. No correlation was found between Kras mutations and microbiota changes or Wif-1 methylation whenever it was considered in stools, blood or urines.

### Accuracy of screening tests to identify advanced neoplasia

The multivariate ORs for each parameter for the presence of advanced neoplasia are shown in Table 4. In a multivariate model adjusted for age, male gender [OR = 2.02; CI95% (1.07–3.82), p = 0.03], history of polyps [OR = 0.24; CI95% (0.11–0.55), p = 0.001] and familial history of CRC [OR = 0.50; CI95% (0.27–0.95), p = 0.03) were independently associated with the presence of advanced neoplasia.

**Table 4:** Factors associated with advanced colorectal neoplasia and a comparison of the accuracies of five multivariate models.

	Model 1: Age + gFOBT		Model 2: Age + Urine or serum Wif-1 methylation test		Model 3: Age + gFOBT + Urine or serum Wif-1 methylation test		Model 4: Age + fecal microbiota dysbiosis		Model 5: Age + gFOBT + fecal microbiota dysbiosis	
Variables	Adjusted OR* (95%CI)	*p†*	Adjusted OR* (95%CI)	*p†*	Adjusted OR* (95%CI)	*P†*	Adjusted OR* (95%CI)	*p†*	Adjusted OR* (95%CI)	*p†*
Age (±1 year)	1.11 (1.07–1.15)	<0.001	1.14 (1.09–1.19)	<0.001	1.14 (1.09–1.20)	<0.001	1.11 (1.07–1.15)	<0.001	1.11 (1.07–1.15)	<0.001
gFOBT	8.07 (3.40–19.17)	<0.001	-	-	4.29 (1.46–12.61)	0.008	-	-	7.27 (3.01–17.6)	<0.001
Urine and/or serum Wif-1 methylation test	-	-	67.1 (18.7–240.22)	≤0.001	53.2 (14.41–196.3)	<0.001	-	-	-	-
*Bacteroides/Prevotella* group (+1 SD)	-	-	-	-	-	-	1.81 (1.14–2.86)	0.01	1.50 (0.94–2.41)	0.09
Calibration and discrimination										
C-Statistic [CI] ‡ (*p* value)	0.83 [0.77–0.88]		0.90 [0.84–0.94] (0.02)		0.91 [0.87–0.95] (≤0.001)		0.79 [0.73–0.85] (0.49)		0.83 [0.78–0.89] (0.44)	
Hosmer-Lemeshow Chi^2^ test (*p* value)	77.7 (*0.58*)		60.6 (0.94)		74.3 (0.98)		220.3 (0.52)		218.1 (0.54)	
Net Reclassification Index (*p* value)	-		Not applicable		28.2 (p = 0.002, vs. model 1)		Not applicable		0 (p = 0.83, vs. model 1)	

OR: Odd ratio; CI, confidence interval SD: standard deviation; CRC: colorectal cancer; gFOBT: guaiac faecal occult blood test; NS: non significant *multivariate logistic regression adjusted for all the variable of the column and age; † Wald test; ‡ CI of C-Statistic was estimated by; ¶ Net Reclassification Index indicated the proportion of patients correctly classified (in the control or case group) when adding a screening test compared to the classification obtained with model 1.

When the age and the results of the FOBT were considered in the same model (model 1), a positive FOBT was independently associated with advanced neoplasia. In a similar model with the results of the urine or serum Wif-1 methylation test (model 2), a positive Wif-1 methylation test was strongly associated with advanced neoplasia (lower bound of 95% CI: 18.7). Adjustment with both FOBT and urine or serum Wif-1 methylation test (model 3) showed that the urine or serum Wif-1 methylation test remained strongly associated with advanced neoplasia (lower bound of 95%CI: 14.41) whereas the FOBT was no longer significant.

When age and fecal microbiota dysbiosis were considered, only the *Bacteroides/Prevotella* group was associated with advanced neoplasia (model 4). When the fecal microbiota dysbiosis and the results of the FOBT were considered in the same model (model 5), only the FOBT was associated with advanced neoplasia.

### Contribution of each model to advanced neoplasia prediction

The goodness of fit of the prediction model that only included age and FOBT was adequate (*p* value of the Hosmer Lemeshow statistic  = 0.58). As shown in Table 4, the accuracy (assessed by C-statistic) of that model was significantly lower than the urine and/or serum Wif-1 methylation test (model 2 increased the C-statistic of 8.6%). This improvement (assessed by C-statistic and Net Reclassification Improvement Index) was significantly higher in the model that combined the urine and/or serum Wif-1 methylation test and the FOBT (model 3 increased the C-statistic of 9.8% and the NRI of 22.8%). Testing for fecal microbiota dysbiosis (model 4) had accuracy similar to the FOBT. Combining FOBT and testing for fecal microbiota dysbiosis did not increase the latter accuracy of the model (model 5 did not increase the C-statistic and the NRI).

## Discussion

CRC is a major cause of mortality and an economic burden in developed countries. The attempt to reduce the mortality of the disease has led public health systems to give priority to mass screenings using FOBT in asymptomatic individuals. Indeed, it has been demonstrated that FOBTs reduced CRC-associated mortality [7], [8], [9], [10]. Although immunochemical measurement of hemoglobin has been shown to be more sensitive than FOBT with similar specificity, the large numbers of individuals who are reluctant to perform the fecal test may limit its use. The effectiveness of such strategies mainly depends on the acceptance and the cost-effectiveness of the screening method. In this setting, blood and urine testing could be used in individuals potentially reluctant to submit to stool testing.

In this study, we compared the diagnostic performance of methylated Wif-1, a secreted antagonist of the Wnt signaling pathway, as a biomarker of advanced colorectal neoplasia in stool, serum and urine samples. We also included clinical characteristics in the screening process and performed prospective FOBTs as a gold standard. We found that methylated Wif-1 had the highest diagnostic performance to predict advanced neoplasia compared to clinical characteristics, FOBTs and microbiota changes, alone or in combination. This finding is of great interest because a significant number of patients decline to perform the FOBT [31].

Thus, for the FOBT performance in the present study, Wif-1 was used to overcome the limitations of FOBT because of its similar diagnostic performance, with 47.8% sensitivity and 97.5% specificity. Colorectal cancer neoplastic cells have proven to be a large paradigm due to the heterogeneity of the molecular pathways implicated in their development [32], [33], [34], [35]. In most cases, the initial genomic alteration of the *APC/Wnt* signaling pathway is followed by the accumulation of additional driver mutations [36]. This relationship has led researchers to believe that a single DNA marker may not be a valuable marker for CRC screening. In our study, the screening for Kras mutations in stool samples has confirmed this dogma by showing very specific but very poorly sensitive results. The use of a fecal DNA panel of 21 gene mutations showed higher sensitivity than the FOBT but has a major cost limitation [12]. Detection of aberrant methylation has recently been shown to be more sensitive than detection of gene mutation [37]. Furthermore, aberrant methylations could also be detected in circulating DNA, such as that found in stool, serum, urine and other body fluids [39]. In this study, we show that the Wif-1 methylation test is an accurate marker of advanced colorectal neoplasia in an initial set of evaluations. Furthermore, we also demonstrate that either urine or blood samples could be used to determine the methylation level. In particular, this marker can be used in individuals who are reluctant to perform stool tests. Notably, performance of FOBT disclosed a lower sensitivity than previously reported. This difference could be explained by the choice of a primary outcome that have included patients with advanced neoplasm which is much more relevant in clinical practice and by the study population that have included patients with an average or high-risk of CRC. This difference could be explain by the extension of the outcome to patients with advanced neoplasm which is much more reliable in clinical practice and by the study population including patients with an average to high risk of CRC. However, extending such a test to the mass screening programs requires further validation in asymptomatic individuals as well as cost effectiveness analyses in a simulation model as previously reported [38].

In addition, to improve the accuracy of biological tests for the prediction of neoplasias during colonoscopies, we investigated markers in microbiota, which is becoming a new field for human disease studies. The contribution of the intestinal microbiota to CRC tumorigenesis is now fully accepted [39]. High throughput sequencing techniques for the human microbiome have been used to investigate changes in the bacterial phylogenetic core in normal individuals and in CRC patients, in either stool or mucosa associated microbiota [21], [22]. Several bacteria groups, i.e., *Bacteroides/Prevotella* [21], *Fusobacterium*, *Faecalibacterium* and particularly *Coriobacteridae* and *Roseburia species* [22], have been shown to be related to CRC, and experimental models have supported this causality hypothesis [24]. *Fusobacterium nucleatum* strains have been shown to promote colorectal carcinogenesis upon invasion of the epithelial cells by producing genotoxin-related direct DNA damage or by inducing inflammation [23], [40], [41]. In the present study, we confirmed a specific dysbiosis associated with advanced colorectal neoplasia characterized by a higher density of species belonging to the *Bacteroides/Prevotella* group. However, this result did not improve the screening performance of the FOBT or the Wif-1 testing. Future studies using deeper sequencing methods and/or metagenomic approaches are warranted to enable a screening tool based on fecal microbiota.

## Conclusion

In this study, the detection of methylated Wif-1 in stool, urine or serum samples has a higher diagnosis accuracy to detect advanced neoplasia compared to FOBT (0.90 [0.84–0.94] vs. 0.83 [0.77–0.88], p = 0.02). Further studies are needed to investigate whether the changes in the microbiota could be biomarkers of advanced colorectal neoplasia, especially using candidate bacteria and metagenomic studies. Serum and urine Wif-1 methylation tests could be used in individuals reluctant to undergo stool testing and should be now evaluated in the context of screening.

## Supporting Information

Figure S1 **Efficiency of primers of target genes (Wif1, ALX-4 and Vimentin) and housekeeping gene (Albumin BSP) used for methylation quantification using serial dilutions of methylated modified DNA control.** For each data point three independent analysis were performed. The equation of the linear regression curve as well as the correlation factor are indicated on each graph.
(DOC)

Table S1 **Methylated gene-targeted primers and probes.**
(DOCX)

Table S2 **Group and species-specific 16S rRNA gene-targeted primers and probes.**
(DOCX)

Table S3 **Kras mutation primers and probes.**
(DOC)
