# Supplementary material for: The Detection of the Methylated Wif-1 Gene Is More Accurate than a Fecal Occult Blood Test for Colorectal Cancer Screening
Source: PLoS One. 2014 Jul 15;9(7):e99233. doi: 10.1371/journal.pone.0099233 (PMC4099003; doi:10.1371/journal.pone.0099233)
Supplement: Figure S1 — Efficiency of primers of target genes (Wif1, ALX-4 and Vimentin) and housekeeping gene (Albumin BSP) used for methylation quantification using serial dilutions of methylated modified DNA control. For each data point three independent analysis were performed. The equation of the linear regression curve as well as the correlation factor are indicated on each graph. (DOC) [file pone.0099233.s001.doc]

**Figure S1:** Efficiency of primers of target genes (Wif1, ALX-4 and Vimentin) and housekeeping gene (Albumin BSP) used for methylation quantification using serial dilutions of methylated modified DNA control. For each data point three independent analysis were performed. The equation of the linear regression curve as well as the correlation factor are indicated on each graph.

y = **- 3,639**x + 24,24

24

26

28

30

32

34

36

-

3

-

2,5

-

2

-

1,5

-

1

-

0,5

0

0,5

Cp

Log(Qt)

**ALX-4**

y = **- 3,506**x + 24,78

24

26

28

30

32

34

36

-

3

-

2,5

-

2

-

1,5

-

1

-

0,5

0

0,5

Cp

Log(Qt)

**Wif-1**

y = **- 3,499**x + 27,61

27

29

31

33

35

37

39

-

3

-

2,5

-

2

-

1,5

-

1

-

0,5

0

0,5

Cp

Log(Qt)

**Vimentin**

y = **- 3,128**x + 27,96

27

29

31

33

35

37

39

-

3

-

2

-

1

0

1

Cp

Log(Qt)

**Albumin BSP**

Cp: cycle quantification; Qt : concentration.
