# Supplementary material for: The Detection of the Methylated Wif-1 Gene Is More Accurate than a Fecal Occult Blood Test for Colorectal Cancer Screening
Source: PLoS One. 2014 Jul 15;9(7):e99233. doi: 10.1371/journal.pone.0099233 (PMC4099003; doi:10.1371/journal.pone.0099233)
Supplement: Table S1 — Methylated gene-targeted primers and probes. (DOCX) [file pone.0099233.s002.docx]

**Table S1: Methylated** g**ene-targeted primers and probes.**

| ***Target gene*** | | ***Primer***  ***and probe*** | ***Sequence 5’- 3’*** |  |
| --- | --- | --- | --- | --- |
| *Wif-1* | Wif-1 S | | TTT GAC GGC GTT AGG TTG C | |
|  | Wif-1 AS | | ACT ACT CAA AAC CTC CTC GCT ACC | |
|  | **Wif-1 met (6Fam)** | | CGG TAC GAG GAG TTT T | |
| *ALX-4* | ALX-4 S | | GTT AAG GCG CGC GGT G | |
|  | ALX-4 AS | | CGC AAA TCT CAA CAT TCA TAC CTA A | |
|  | **ALX-4 met (6Fam)** | | TCG TTC GTC GTT TGC | |
| *Vimentine* | Vimentin S | | GTT TTA GTC GGA GTT ACG TGA TTA CG | |
|  | Vimentin AS | | AAC ACG CTA CTC CGC AAA CG | |
|  | **Vimentin met (6Fam)** | | CGT ATT TAT AGT TTG GGT AGC G | |
| *Albumin* | Albumin BSP S | | GGG ATG GAA AGA ATT TTA TGT T | |
|  | Albumin BSP AS | | AAA CAA ACT AAC CCC AAA TTC T | |
|  | **Albumin BSP Probe (6Fam)** | | AGG TTA AGG GTT TTT ATA ATT TA | |

Primers and probes were constructed to target Wif-1, Vimetin, ALX-4 and albumin genes. The first primer listed is the forward PCR primer, the second is the reverse PCR primer and the third is the Taq-man® primer . Methods have been previously described by Eads et al. [25].
