# Supplementary material for: The Detection of the Methylated Wif-1 Gene Is More Accurate than a Fecal Occult Blood Test for Colorectal Cancer Screening
Source: PLoS One. 2014 Jul 15;9(7):e99233. doi: 10.1371/journal.pone.0099233 (PMC4099003; doi:10.1371/journal.pone.0099233)
Supplement: Table S2 — Group and species-specific 16S rRNA gene-targeted primers and probes. (DOCX) [file pone.0099233.s003.docx]

**Table S2**: **Group and species-specific 16S rRNA gene-targeted primers and probes.**

| ***Target organism*** | ***Primer***  ***and probe*** | | ***Sequence 5’- 3’*** | |  |
| --- | --- | --- | --- | --- | --- |
| *All-bacteria* **(*)** | | F_Bact 1369 | | CGG TGA ATA CGT TCC CGG | |
|  | | R_Prok1492 | | TAC GG**C** TAC CTT GTT ACG ACT T | |
|  | | **P_TM1389F** | | **6FAM-CTT GTA CAC ACC GCC CGT C** | |
| *C. leptum* | | F_Clept 09 | | CCT TCC GTG CCG SAG TTA | |
|  | | R_Clept 08 | | GAA TTA AAC CAC ATA CTC CAC TGC TT | |
|  | | **P-Clep 01** | | **6FAM-CAC AAT AAG TAA TCC ACC** | |
| *Bifidobacterium* | | F_Bifid 09c | | CGG GTG AGT AAT GCG TGA CC | |
|  | | R_Bifid 06 | | TGA TAG GAC GCG ACC CCA | |
|  | | **P_Bifid** | | **6FAM-CTC CTG GAA ACG GGT G** | |
| *C. coccoides* | | F_Ccoc 07 | | GAC GCC GCG TGA AGG A | |
|  | | R_Ccoc 14 | | AGC CCC AGC CTT TCA CAT C | |
|  | | **P_Erec482(*)** | | **VIC-CGG TAC CTG ACT AAG AAG** | |
| *Bacteroides/* | | F_Bacter 11 | | CCT WCG ATG GAT AGG GGT T | |
| *Prevotella* | | R_Bacter 08 | | CAC GCT ACT TGG CTG GTT CAG | |
|  | | **P_Bac303(*)** | | **VIC-AAG GTC CCC CAC ATT G** | |
| *E. coli* | | E.coli F | | CAT GCC GCG TGT ATG AAG AA | |
|  | | E.coli R | | CGG GTA ACG TCA ATG AGC AAA | |
| *Lactobacillus/* | | F_Lacto 05 | | AGC AGT AGG GAA TCT TCC A | |
| *Leuconostoc/ Pediococcus* | | R_Lacto 04 | | CGC CAC TGG TGT TCY TCC ATA TA | |
| *F. prausnitzii* | | Fprau 07 | | CCA TGA ATT GCC TTC AAA ACT GTT | |
|  | | Fprau 02 | | GAG CCT CAG CGT CAG TTG GT | |

(*) modified from reference

Primers and probes were constructed to target all dominant and subdominant bacterial species in accordance to the pyrosequencing results. Methods have been previously described [26]. Probe sequences are in bold.
