# Supplementary material for: The Detection of the Methylated Wif-1 Gene Is More Accurate than a Fecal Occult Blood Test for Colorectal Cancer Screening
Source: PLoS One. 2014 Jul 15;9(7):e99233. doi: 10.1371/journal.pone.0099233 (PMC4099003; doi:10.1371/journal.pone.0099233)
Supplement: Table S3 — Kras mutation primers and probes. (DOC) [file pone.0099233.s004.doc]

**Table S3: Kras mutation primers and probes.**

| ***Target gene*** | | ***Primer***  ***and probe*** | ***Sequence 5’- 3’*** |  |
| --- | --- | --- | --- | --- |
| *Kras wild (exon 12)* | Forward | | AGG CCT GCT GAA AAT GAC TGA ATA T | |
|  | Backward | | GCT GTA TCG TCA AGG CAC TCT T | |
|  | **VIC** | | CCT ACG CCA CCA GCT | |
| *Kras wild (exon 12)2* | Forward | | AGG CCT GCT GAA AAT GAC TGA ATA T | |
|  | Backward | | GCT GTA TCG TCA AGG CAC TCT T | |
|  | **VIC** | | CTA CGC CAC CAG CTC | |
| *Kras mutant (exon 12)* | Forward | | AGG CCT GCT GAA AAT GAC TGA ATA T | |
|  | Backward | | GCT GTA TCG TCA AGG CAC TCT T | |
|  | **FAM** | | CTA CGC CAG CAG CT | |
| *Kras mutant (exon 13)* | Forward | | AGG CCT GCT GAA AAT GAC TGA ATA T | |
|  | Backward | | GCT GTA TCG TCA AGG CAC TCT T | |
|  | **FAM** | | ACG CCA ACA GCT C | |

Primers and probes were constructed to target codon 12 and 13 of the Kras gene. In all cases, the first primer listed is the forward PCR primer, the second is the reverse PCR primer and the third is the TaqMan® probe (Fam dye was used for mutant allele and Vic dye for the wild one)
